# Localized multiple malignant epithelioid peritoneal mesotheliomas arising from the hepatoduodenal ligament and diaphragm: a case report

**DOI:** 10.1186/s13256-019-2008-9

**Published:** 2019-03-18

**Authors:** Takashi Miyata, Yuta Fujiwara, Koji Nishijima, Fumio Futagami, Takashi Nakamura, Hiroyuki Takamura

**Affiliations:** 1 Department of Surgery, Japanese Red Cross Kanazawa Hospital, Kanazawa, Ishikawa 921-8162 Japan; 2 Department of Gastroenterological Surgery, Division of Cancer Medicine Graduate School of Medicine, Kanazawa University, Kanazawa, Ishikawa 920-8641 Japan; 3 Department of General and Digestive Surgery, Kanazawa Medical University Hospital, 1-1 Daigaku, Uchinada, Kahoku, Ishikawa 920-0293 Japan

**Keywords:** Localized mesothelioma, Malignant peritoneal mesothelioma, Angiography, Case report

## Abstract

**Background:**

Malignant peritoneal mesothelioma is a rare aggressive tumor of the peritoneum. We report a rare case of resection of multiple localized malignant peritoneal mesotheliomas.

**Case presentation:**

A 55-year-old Japanese woman was admitted to our hospital because liver tumors were detected by abdominal ultrasonography during a screening examination. Blood examination findings, including tumor makers, were within normal ranges. She had no evidence of exposure to asbestos. Computed tomography showed four hypervascular, round liver tumors, one in the lateral liver segment adjacent to the hepatic hilus, and the other three on the liver surface. Computed tomography angiography revealed that the tumor in the lateral segment had strong enhancement and was fed from the left gastric artery. In contrast, the other tumors showed no enhancement, and were fed from the right inferior phrenic artery. Abnormal accumulation was identified in the four tumors only with ^18^F-fluorodeoxyglucose positron emission tomography. It was very difficult to obtain a definitive preoperative diagnosis, but surgical resection was performed because we considered potential malignancy. Laparotomy revealed the principal site of the tumor in the lateral segment was on the hepatoduodenal ligament, and all other tumors were on the diaphragm. A left lobectomy and partial diaphragmatic resection were performed. The final pathological diagnosis was multiple malignant epithelioid mesotheliomas. Our patient has had no recurrence for 20 months postoperatively.

**Conclusions:**

In general, malignant peritoneal mesotheliomas are classified as diffuse tumors, which are often unresectable and have a poor prognosis. However, early diagnosis and treatment, particularly with the localized type, as in our patient, could lead to long-term survival of the patient. We recommend that multiple malignant epithelioid mesotheliomas be included in the differential diagnosis for patients with subcapsular hepatic tumors.

## Introduction

Malignant peritoneal mesothelioma (MPM) is a rare but rapidly fatal malignancy. MPM is difficult to diagnose because clinical symptoms and findings are nonspecific. The median survival ranges from 5 to 12 months, mainly because of a lack of effective treatment [1]. We present a case of a 55-year-old Japanese woman without asbestos exposure to illustrate the rarity and difficulty of making a diagnosis of localized MPM. This case report focuses on the image findings of the tumors, which were similar to those of hepatic tumors, but computed tomographic (CT) angiography showed that the tumors were actually primary peritoneal tumors. Patients with MPM, particularly those with the localized type, as in our patient, may be cured by early diagnosis and treatment by surgical excision.

## Case presentation

**Fig. 1:**
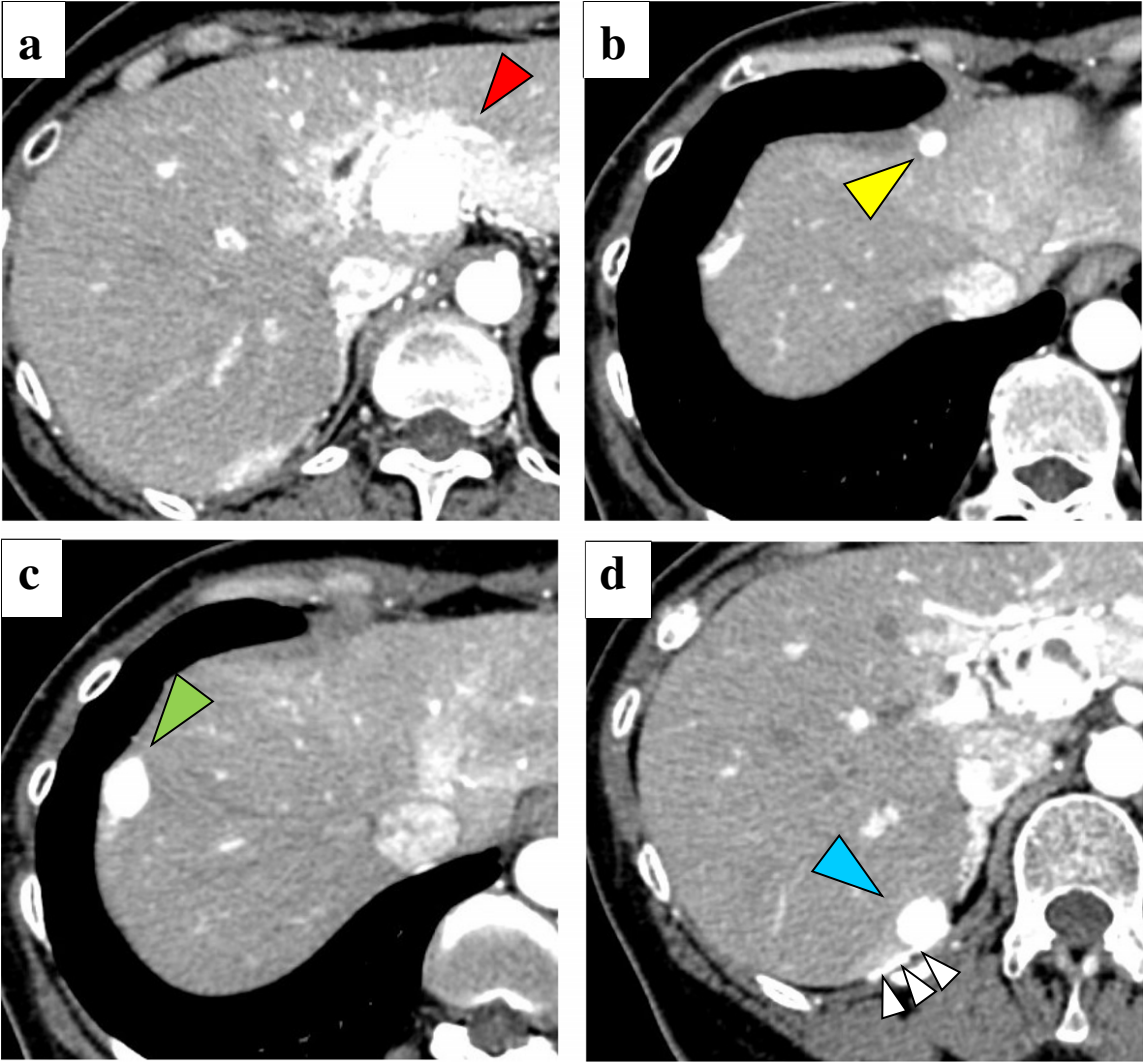
Enhanced abdominal computed tomography showing a hypervascular tumor measuring 32-mm diameter (**a**, *red arrow*) in contact with the lateral liver segment. A 7-mm tumor in segment 4 (**b**, *yellow arrow*), 15-mm tumor in segment 8 (**c**, *green arrow*), and 17-mm tumor in segment 7 (**d**, *blue arrow*) are also seen. All tumors are positioned on the liver periphery. Moreover, the tumor in segment 7 is continuous with the diaphragm and has the same high density of contrast medium (*white arrows*)

**Fig. 2:**
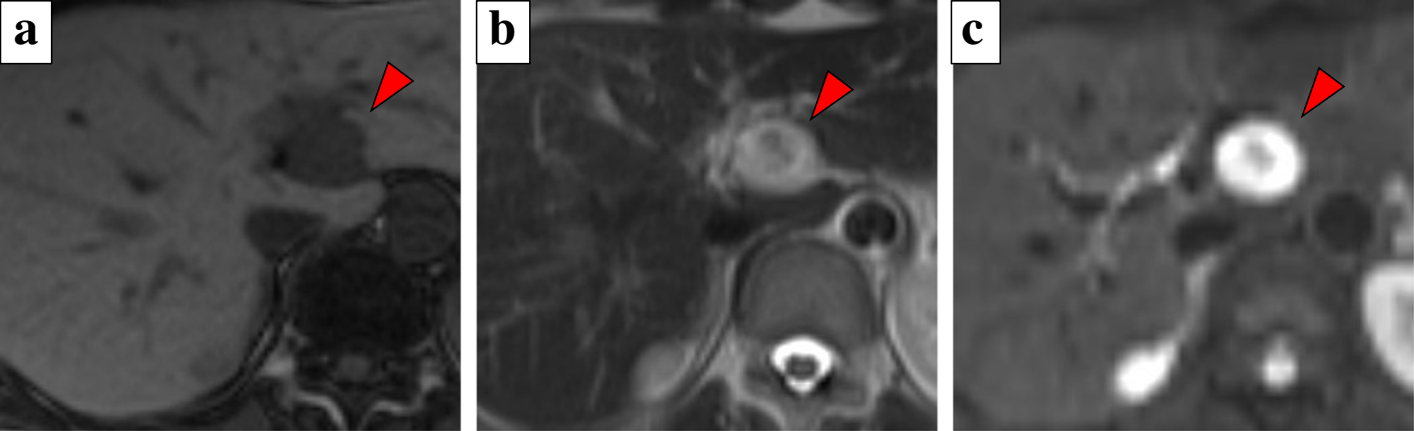
Magnetic resonance images showing a tumor with lower intensity compared with the liver on T1-weighted images (**a**, *red arrowhead*), and with higher intensity than the liver on T2-weighted and diffusion-weighted images (**b** and **c**, *red arrowhead*)

A 55-year-old Japanese woman was referred to our hospital because of hepatic tumors detected by abdominal ultrasonography during a screening examination. Her medical and family history was unremarkable; her occupational and residential history showed no apparent exposure to asbestos. She received no medical drugs, and neither smoked tobacco nor drank alcohol. On admission, her general status was unremarkable; her temperature was 36.2 °C and her blood pressure was 110/70 mmHg with a regular heart rate of 80/minute. A physical examination revealed no abnormal findings; neurological examinations were unremarkable. All laboratory data, including levels of the serum tumor markers carcinoembryonic antigen, cancer antigen 19-9, alpha fetoprotein, protein induced by vitamin K absence or antagonist-II, and cancer antigen 125 were within normal ranges (Table 1). An abdominal enhanced CT scan revealed four hypervascular, round hepatic tumors. One tumor, which was 32 mm in diameter, was in contact with the lateral liver segment and the hepatic hilus; the other three tumors were in contact with the diaphragm and measured 7 mm in segment 4, 17 mm in segment 7, and 15 mm in segment 8 (Fig. 1a–d). There was no pleural effusion or ascites, and no other nodular lesions in her chest or abdomen were observed. Similar to the CT findings, magnetic resonance imaging (MRI) revealed a tumor of lower intensity than the liver on T1-weighted images, and with higher intensity than the liver on T2-weighted and diffusion-weighted images (Fig. 2a–c). Upper and lower gastrointestinal endoscopic examination findings were normal. We hypothesized that the tumor arose from her liver, and diagnosed our patient as having multiple hepatic hemangiomas, at first. However, imaging findings were not typical of hepatic tumor: all tumors were on the edge of the liver and the tumor margins were clear. Moreover, the tumor in segment 7 appeared continuous with the diaphragm with similar high-density contrast medium (Fig. 1d). We then performed CT angiography to obtain further details.

**Table 1:** Results of laboratory tests on admission

Variables	Results of laboratory tests
WBC (×10^3^/μl)	5.84
RBC (× 10^6^/μl)	3.93
Hemoglobin (g/dl)	13.9
Hematocrit (%)	40.1
Platelet (× 10^4^/μl)	20.1
Total protein (g/dl)	7.9
Albumin (g/dl)	4
CRP (mg/dl)	0.1
BUN (mg/dl)	9.6
Creatinine (mg/dl)	0.59
Na (mEq/l)	139
K (mEq/l)	3.9
Cl (mEq/l)	107
AST (IU/l)	21
ALT (IU/l)	20
ALP (IU/l)	171
LDH (IU/l)	176
Total bilirubin (mg/dl)	0.4
Amylase (IU/l)	85
CEA (ng/ml)	0.9
CA19-9 (U/ml)	1.0
AFP (ng/ml)	2.5
PIVKA-II (mAU/ml)	17
CA125 (U/ml)	20

*AFP* alpha fetoprotein, *ALP* alkaline phosphatase, *ALT* alanine aminotransferase, *AST* aspartate aminotransferase, *BUN* blood urea nitrogen, *CA125* cancer antigen 125, *CA19-9* cancer antigen 19-9, *CEA* carcinoembryonic antigen, *CRP* C-reactive protein, *LDH* lactate dehydrogenase, *PIVKA-II* protein induced by vitamin K absence or antagonist-II, *RBC* red blood cell, *WBC* white blood cell

**Fig. 3:**
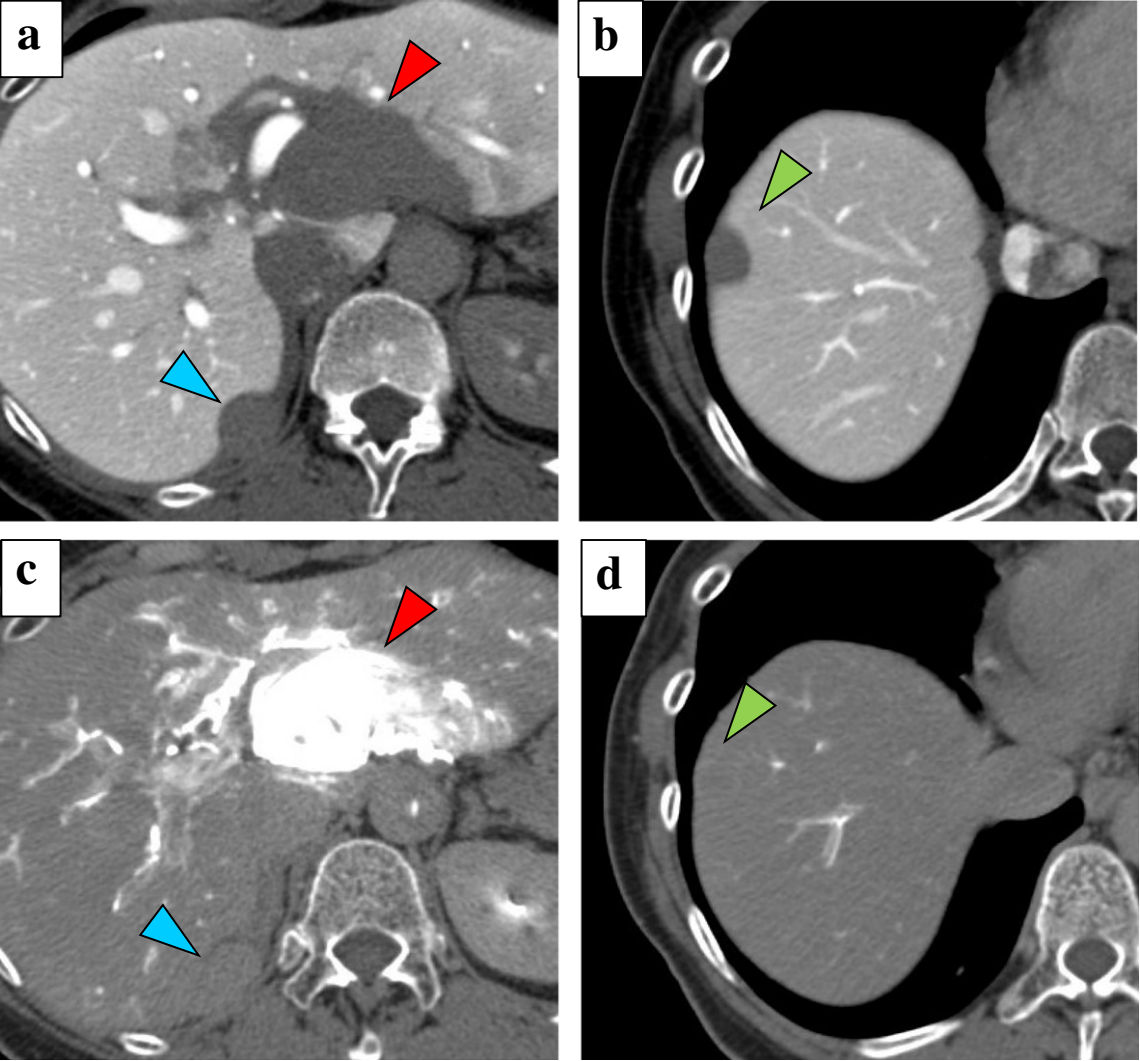
Computed tomography (CT) during arterial portography showed enhancement in none of the tumors (**a**, **b**), but CT during hepatic arteriography revealed strong enhancement in the tumor in the lateral segment. In contrast, the remaining three tumors showed no enhancement (**c**, **d**) (*red arrow*, the tumor in the lateral segment; *blue arrow*, the tumor in segment 7; *green arrow*, the tumor in the in segment 8)

**Fig. 4:**
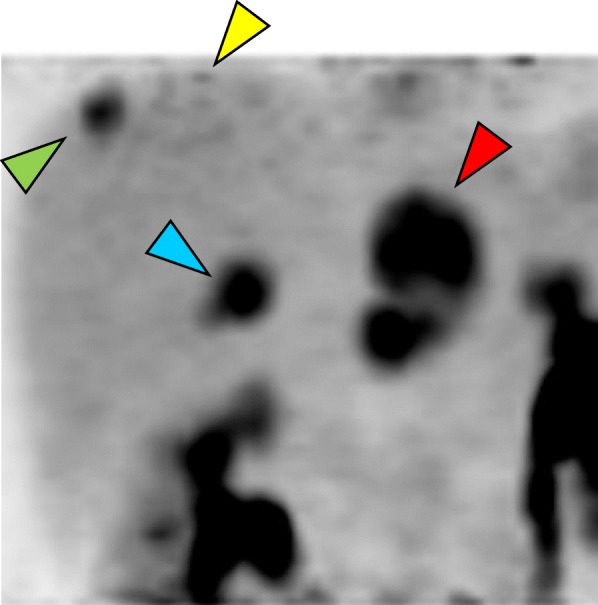
Fluorodeoxyglucose-positron emission tomography showing increased fluorodeoxyglucose uptake (*red arrow*, lateral segment; *yellow arrow*, segment 4; *green arrow*, segment 8; *blue arrow*: segment 7)

**Fig. 5:**
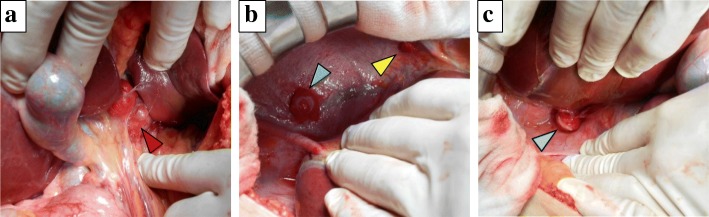
Operative findings showing one tumor located between the liver and hepatorenal ligament (**a**; *red arrow*) three other tumors located on the diaphragm (**b**; *green* and *yellow arrow*, **c**; *blue arrow*)

**Fig. 6:**
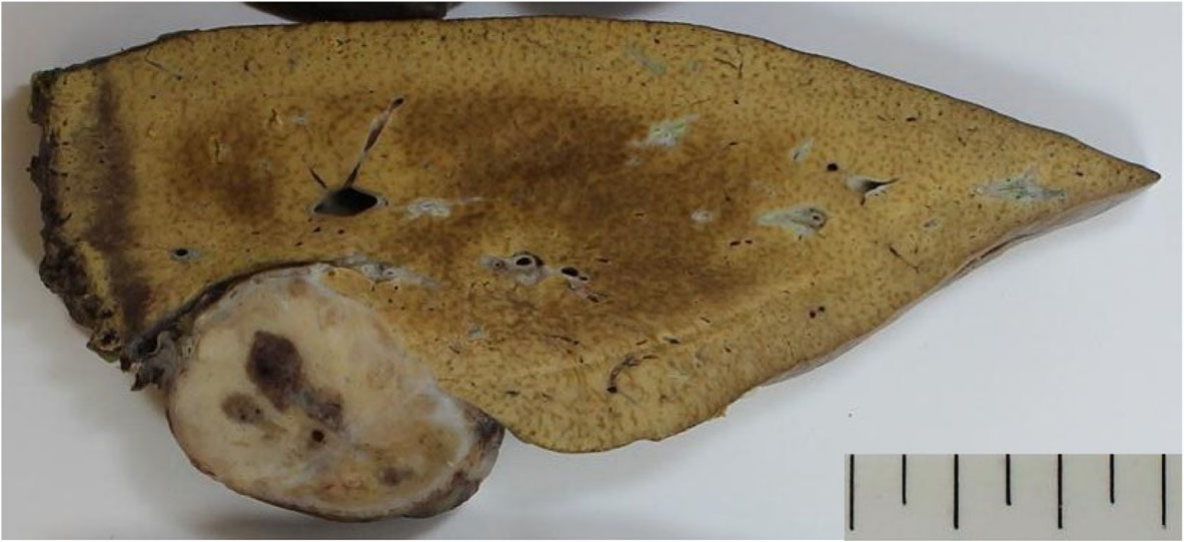
On gross examination, the tumor resected by left lobectomy measures 3.6 cm in diameter, and the surface and demarcation between the tumor and liver are smooth

**Fig. 7:**
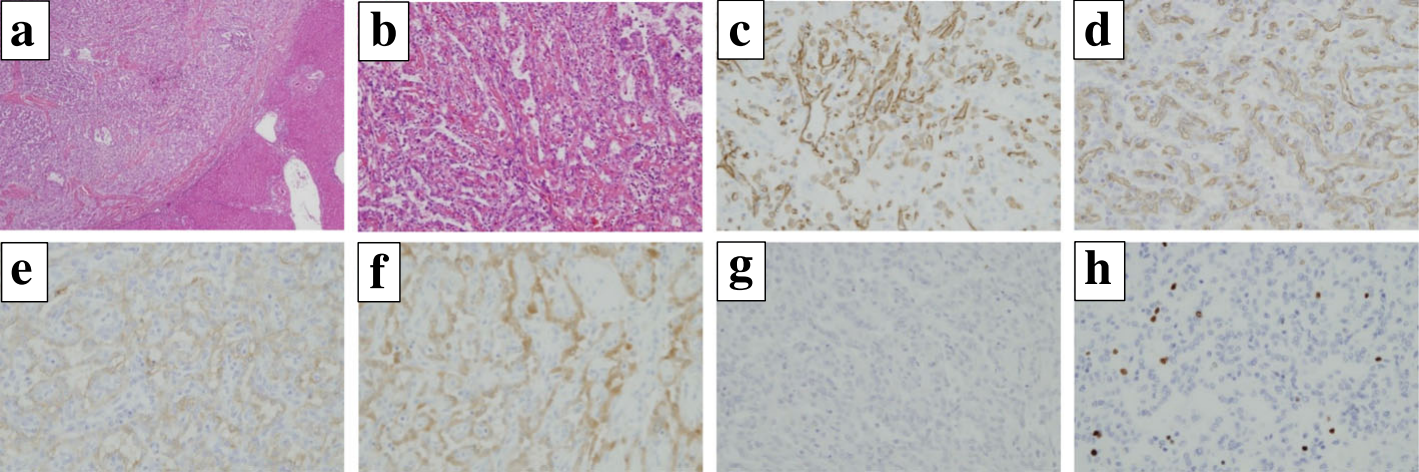
Histopathological findings showing that tumor cells increased in a papillary form containing an epithelial pattern (**a**, original magnification × 30; **b**, original magnification × 100; all, hematoxylin and eosin stain). Tumor cells stained positively for CD31 (**c**), CD34 (**d**), D2-40 (**e**), and calretinin (**f**), and negatively for p53 (**g**). The Ki-67 labeling index was < 20% (**h**) (**c**–**h**, original magnification × 100)

CT findings during arterial portography showed enhancement in none of the tumors (Fig. 3a and b), but CT during hepatic arteriography revealed strong enhancement in the tumor in the lateral segment and that this tumor was fed by the left gastric artery. In contrast, the remaining three tumors had no enhancement and were fed by the right inferior phrenic artery (Fig. 3c and d). Imaging findings indicated that the liver was not the origin of these tumors. Moreover, all tumors exhibited increased uptake of ^18^F-fluorodeoxyglucose, with a standardized uptake value of 7.8 with positron emission tomography (PET)-CT (Fig. 4). We considered these tumors to be peritoneal malignant tumors, preoperatively, and performed surgical resection. Laparotomy revealed that the tumor in the lateral segment was clearly in contact with the hepatoduodenal ligament, and that the other three tumors were on the diaphragm (Fig. 5a–c). We performed tumorectomy with left liver lobectomy (Fig. 6) and partial diaphragmatic resection. Histopathological and immunohistochemical examinations of the resected specimens were positive for CD34, CD31, D2-40, and calretinin, which confirmed epithelioid mesothelioma (Fig. 7a–h). Our patient was not given any medication except a general drip infusion and prophylactic antibiotics; she was discharged 10 days after surgery without complications. CT and PET-CT revealed no recurrence 20 months after surgery without adjuvant therapy.

## Discussion

In the present case report, we present a rare case of MPM which is an aggressive tumor and very difficult to diagnose. Moreover, the multiple localized MPMs seen in our patient are extremely rare and, to the best of our knowledge, have not been addressed in the literature. However, our patient was successfully diagnosed as having peritoneal malignant tumor preoperatively and treated with surgical resection.

MPM is a rare neoplastic condition that arises from the serosal membranes of the abdominal cavity and is classified as either diffuse or localized [2]. MPM presents in the vast majority of patients as the diffuse type as multiple peritoneal nodules, sometimes with dense adhesions, and is almost always associated with ascites. By contrast, localized MPM is much less common, and usually appears as a rare solitary circumscribed nodular tumor [3].

Diagnosis of multiple localized MPMs can be very difficult preoperatively and should be considered in any patient with subcapsular hepatic tumors. MPM often presents as an incidental finding or with nonspecific symptoms. A physical examination does not reveal abnormalities until the disease has advanced significantly, and standard laboratory and radiographic studies are often inconclusive [4, 5]. Ascites is often absent, which makes the diagnosis more difficult, and exposure to asbestos as a cause of localized MPM is not usual, while many cases of diffuse MPM are associated with asbestos exposure [1, 3]. In some patients, diagnostic imaging such as CT, MRI, and positron emission tomography may provide useful information, but these are not sufficient to establish a definitive diagnosis of mesothelioma. In most patients, the definitive diagnosis is obtained by laparoscopy or open surgery with biopsy to obtain histological examination along with immunocytochemical procedures, as in our patient, and others [6–8].

This case report highlights aspects of diagnosis and management of a very rare disease that presents with atypical characteristics in preoperative imaging. At first, the disease presentation resembles extrahepatic tumor growth, mimicking hepatic multiple hemangiomas. However, all the tumors in our patient were located on the edge of the liver, and the density of one of the tumors matched the density of slight peritoneal thickening of the diaphragm, suggesting a tumor arising from the peritoneal wall. The identification of feeding vessels with CT angiography proved key to the diagnosis of peritoneal tumor in our patient. We considered MPM as one of the preoperative differential diagnoses, and started early treatment. The prognosis for patients with MPM is poor, and the management of localized MPM is controversial. However, in the largest series to date, Allen and Cagle *et al*. described 23 cases of localized malignant mesothelioma, and 50% of patients were alive after several years of follow-up, in contrast to those with diffuse malignant mesothelioma [3]. Surgery is the first treatment choice, and complete cure is expected postoperatively for localized MPM.

Because of its rarity and minimal available treatment information, it is difficult to make a primary diagnosis, and by the time of definitive diagnosis, most patients have wide tumor spread to other organs. However, surgeons should consider a peritoneal tumor in the differential diagnosis, and identification of feeding vessels with contrast angiography or CT angiography as an auxiliary diagnostic method for localized MPM, especially for tumors on the subcapsular liver.

## Conclusions

In general, MPM is a very rare disease and may mimic a benign tumor radiologically. MPM has a poor prognosis; however, early diagnosis and treatment, particularly with the localized type, as in our patient, could lead to long-term survival of the patient. MPM should be included in the differential diagnoses of patients with subcapsular hepatic tumors, and we believe that this case report will be useful for future clinical diagnosis and treatment of the disease.

## Abbreviations

CT: Computed tomography
MPM: Malignant peritoneal mesothelioma
MRI: Magnetic resonance imaging
PET: Positron emission tomography
